# Cold-Brewed Jasmine Tea Attenuates High-Fat Diet-Induced Obesity and Gut Microbial Dysbiosis

**DOI:** 10.3390/nu14245359

**Published:** 2022-12-16

**Authors:** Ang Li, Jin Wang, Xuejiao Zhang, Ruixin Kou, Mengshan Chen, Bowei Zhang, Jingmin Liu, Bo Peng, Yan Zhang, Shuo Wang

**Affiliations:** Tianjin Key Laboratory of Food Science and Health, School of Medicine, Nankai University, Tianjin 300350, China

**Keywords:** cold brew, jasmine tea, high-fat diet, obesity, gut microbiota, mice

## Abstract

Cold-brewed jasmine tea (CB-JT) is regarded to possess characteristic flavors and health benefits as a novel resource of functional tea beverages. To investigate the molecular mechanisms underlying CB-JT-mediated protective effects on obesity, we evaluated the serum biochemistry, histological condition, glucose tolerance, gene expression profile and intestinal microbial diversity in high-fat diet (HFD)-fed mice. Our results demonstrate that cold-brewed jasmine tea can significantly attenuate HFD-induced body weight gain, abnormal serum lipid levels, fat deposition, hepatic injury, inflammatory processes as well as metabolic endotoxemia. CB-JT also modified the microbial community composition in HFD-fed mice and altered the balance to one closely resembled that of the control group. The differential abundance of core microbes in obese mice was reversed by CB-JT treatment, including an increment in the abundance of *Blautia*, *Mucispirillum*, and *Bilophila* as well as a decrease in the abundance of *Alloprevotella*. CB-JT was proved to regulate the mRNA expression levels of lipid metabolism-related genes such as *Leptin*, *Pgc1a Il6*, and *Il1b* in the adipose tissue coupled with *Cyp7a1*, *Lxra*, *Srebp1c*, and *Atgl* in the liver. These findings indicate that cold-brewed jasmine tea might be served as a potential functional tea beverage to prevent obesity and gut microbiota dysbiosis.

## 1. Introduction

Recently, obesity has emerged as an increasingly pivotal global health issue, characterized by dyslipidemia, lipid accumulation, metabolic disorder, low-grade inflammation, and intestinal dysfunction [1]. Obesity has also been linked to multiple metabolic diseases such as diabetes, hyperglycemia, hepatosteatosis, cardiovascular disease, and even behavioral disorders [2]. Increasing studies have indicated that gut microbiota exhibits a critical role in developing/preventing obesity through involvement in nutrient digestion, energy metabolism, and chronic inflammation [3]. Diet has been recently confirmed to modulate the intestine microbial composition and thus affect the course of obesity [4]. Therefore, therapeutic strategies targeting gut microbes by dietary intervention may effectively combat overweight, obesity, and other related metabolic diseases.

Sugary drinks, including ready-to-drink tea beverages, contribute considerably to sugar consumption and overall energy intake, which is closely associated with the onset of obesity [5]. It is thus expected that by 2025, the global sugar-free tea market will continue to proliferate due to rising consumer awareness regarding the health benefits of sugar-free beverages [6]. Meanwhile, a population-based study on functional food has revealed that customers are reluctant to lower their taste expectations for health attributes [7]. Interestingly, the emergence of cold brewing may improve the recognition of unsweetened tea, delivering a novel tea beverage with health attributes and a less bitter taste, as documented for cold-brewed green tea [8]. Cold-brewed tea, originated in Taiwan, is generally brewed with cold water, exhibiting the inherent health advantages of traditional tea coupled with the excellent taste of ready-to-drink beverages. It has been reported that cold-brewed tea has more bioactive ingredients compared with hot-brewed tea [9]. Cold brewing has also been proven to prevent/retard the oxidation process of tea polyphenols, protect nutrient integrity, and maintain premium flavor [10].

Jasmine tea (JT), as a type of Chinese green tea, is commonly processed with jasmine aroma and thus widely acclaimed for its characteristic aroma [11]. In 2019, the total yield and gross output value of the JT industry in China reached 1.14 thousand metric tons and CNY 12.19 billion, respectively, which proved its enormous development potential [12]. JT is also highly valued for its potential health benefits, such as antioxidant effects and sedative properties [13,14]. Although there are several recent studies regarding anti-obesity effects of green tea, black tea, and oolong tea in animal models [15,16,17], the mechanism attributable to the jasmine tea-mediated protective effect against obesity involving the gut microbiome has not yet been elucidated.

Herein, we sought to explore the preventive effect of cold-brewed jasmine tea (CB-JT) on obesity in C57BL/6J mice fed with a high-fat diet (HFD). The objectives of this study were to (1) monitor the effect of CB-JT on the serum lipid level, inflammatory process, hepatic function, glucose intolerance, and gene expression profiling related to lipid metabolism; (2) assess the impact of CB-JT on the pathological symptoms of the liver, colon, and adipose tissue; and (3) evaluate gut microbial diversity with CB-JT intervention, thus exploring the role of gut microbiota in JT-mediated prevention of obesity. Our findings aim to provide a theoretical basis for the development of an alternative functional beverage that prevents obesity and benefits human metabolic health.

## 2. Materials and Methods

### 2.1. Preparation of Cold-Brewed Jasmine Tea

Jasmine tea in this experiment was purchased from Yunbiao Town, Hengxian, Guangxi, China. Briefly, fresh tea was preprocessed and mixed with jasmine, and then reheated, scented, separated, dried, and cooled. CB-JT was obtained by brewing the prepared jasmine tea leaves with 80-fold (*m*/*v*) cold water and steeping for 8 h at 4 °C. The tea infusion was collected prior to further analysis. Tea phenolics and polysaccharides were quantified using the Folin–Ciocalteu method and phenol-sulfuric acid method, respectively [18]. The concentrations of the major catechins, theanine, and gallic acid in the brews were also evaluated in duplicate by high-performance liquid chromatography (HPLC). The experimental program and bioactive contents in CB-JT can be found in Supplementary Material, Table S1.

### 2.2. Experimental Animal Design

Twenty-four male C57BL/6 mice (6 weeks old) purchased from Vital River Laboratories (Peking, China) were housed in a temperature- and humidity-controlled facility (temperature 22 ± 1 °C and humidity 50 ± 10%) and were randomly divided into three groups (*n* = 8) after 1 week adaptation: a normal-chow (NC) group (normal-chow diet containing 10% calories from fat, D12450J, with pure water, an HFD group (HFD containing 60% calories from fat, D12492, plus pure water), and the JT group, fed HFD with unlimited CB-JT supplementation as drinking water. The NC diet and HFD were purchased from Xietong Co., Ltd. (Shanghai, China). Food and water consumption of each group were measured every 2 days, and body weights were documented once a week. All animal care and experiments were in accordance with the institutional guidelines for animal use of Nankai University. The oral glucose tolerance test (OGTT) was further performed after glucose administration at the 8th week. The blood glucose levels from tail nicks were monitored at fasting and 2 h post-glucose gavage, and the area under the curve for each algorithm was computed as well. At the end of the experimental schedule, the fresh fecal pellets and blood samples were separately harvested, and animals were then euthanized by cervical dislocation to obtain liver, adipose and colon tissues for subsequent biochemical and pathological analyses after an overnight fast.

### 2.3. Measurement of Serum Parameters and Hepatic Lipid Profiles

The serum concentration of triglyceride (TG), total cholesterol (TC), low-density lipoprotein cholesterol (LDL-C), high-density lipoprotein cholesterol (HDL-C), glucose as well as the content of aspartate aminotransferase (AST) and alanine aminotransferase (ALT) were quantified using commercial test kits (Nanjing Jiancheng Bioengineering Institute, Nanjing, China). The levels of lipopolysaccharide (LPS), tumor necrosis factor-alpha (TNF-α), and interleukin 6 (IL-6) were evaluated with ELISA kits (Cusabio Biotech Co., Ltd., Wuhan, China).

### 2.4. Hematoxylin and Eosin (H&E) Staining

Fresh adipose, liver and colon tissues were fixed in 10% neutral-buffered formalin, paraffined, embedded, sectioned, H&E stained, and processed for microscopic examination. The adipocyte area was quantified using ImageJ software.

### 2.5. Quantification of Gene Expression

Total RNA from epididymal adipose and liver tissue was extracted using TRIzol Reagent (Ambion, Austin, TX, USA) in line with the manufacturer’s instructions and reverse-transcribed into cDNA using Revert Aid First Strand cDNA Synthesis Kit (Thermo Fisher Scientific, Waltham, MA, USA). The mRNA expression levels of lipid metabolism-related genes were detected using qRT–PCR on the CFX Connect Real-Time System (BIO-RAD, Hercules, CA, USA). The primers used are provided in Table S2, Supplementary Material. Relative quantification was normalized using β-actin control and achieved according to the comparative 2^−ΔΔ^Ct method.

### 2.6. Gut Microbiota Analysis

Metagenomic DNA was extracted from fecal pellets, followed by 16S rRNA amplification of the variable 4 (V4) regions using an Illumina MiSeq platform (Novogene Genomics Technology Co. Ltd., Beijing, China) with the universal primers 341F and 806R. The amplicons were extracted, purified, quantified, and then clustered with the Uparse software, where the sequences with ≥97% similarity were clustered into operational taxonomic units (OTUs). The representative sequences were identified to perform further species taxonomic analysis.

### 2.7. Statistical Analysis

The effects of CB-JT on observed variables were exhibited as means ± SEM and calculated using GraphPad Prism 5 Software (GraphPad, San Diego, CA, USA) and SPSS 20.0 (SPSS Inc., Chicago, IL, USA). The gut microbiota diversity including alpha diversity, beta diversity, gut microbial composition, linear discriminant analysis (LDA) effect size (LEfSe), Spearman’s correlation, redundancy analysis (RDA) and functional prediction was analyzed on the Novogene Bioinformatics platform (Beijing, China). Two-tailed Student’s *t*-tests were used when comparing the statistical differences between two groups, and one-way analysis of variance (ANOVA) test was used to compare among three groups. *p* values < 0.05 were considered statistically significant.

## 3. Results

### 3.1. CB-JT Suppressed HFD-Induced Abnormal Body Weight Gain, Organ Weight and Food Intake

**Figure 1:**
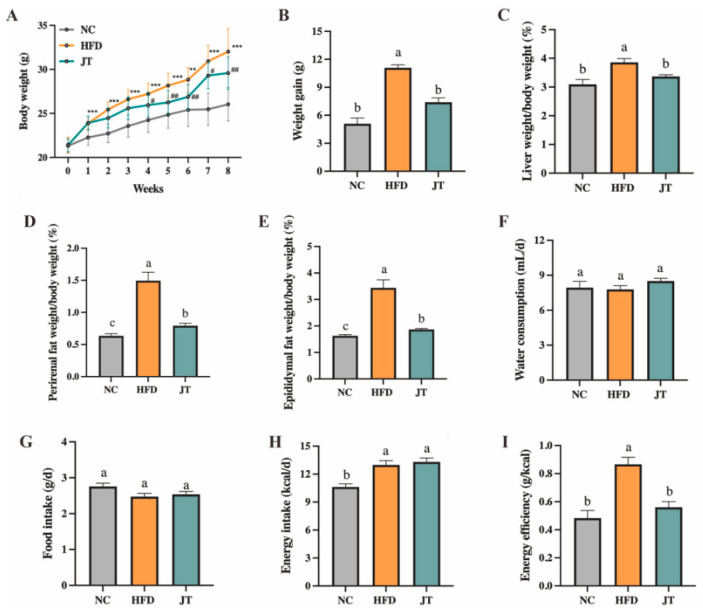
CB-JT suppressed HFD-induced abnormal body weight gain, organ weight and food intake. (**A**) Body weight during the 8-week intervention (*n* = 8). (**B**) Body weight gain. (**C**) Liver weight/body weight. (**D**) Perirenal fat weight/body weight. (**E**) Epididymal fat weight/body weight. (**F**) Water consumption. (**G**) Food intake. (**H**) Energy intake. (**I**) Energy efficiency. NC: normal-chow group, HFD: high-fat diet group, JT: jasmine tea group. Data are expressed as the mean ± SEM. The mean value with different letters indicates significant differences (*p* < 0.05). HFD vs. NC: ** *p* < 0.01, *** *p* < 0.001; JT vs. HFD: # *p* < 0.05, ## *p* < 0.01, ns for no significance.

To evaluate the preventive effect of CB-JT on the obesity process, the obesity-related parameters in HFD-fed mice supplemented with CB-JT for 8 weeks were measured. Figure 1A,B show that starting on the 4th week of HFD feeding, body weights in the JT group were notably lower than those in the HFD group (*p* < 0.05). In comparison with the HFD group, the significant decreases in the perirenal fat indices (perirenal fat weight to body weight ratio), epididymal fat indices (epididymal fat weight to body weight ratio) and liver indices (liver weight/body weight ratio) of the JT group were as shown in Figure 1C–E. No significantly statistical difference in food intake and water consumption among NC, HFD, and JT groups was evident (Figure 1F,G). The higher energy intake resulting from the higher energy density in the HFD group is noted in Figure 1H,I, whereas the significantly elevated energy efficiency (weight gain/energy intake ratio) driven by HFD feeding was reversed by CB-JT intervention with statistical significance (*p* < 0.05).

### 3.2. CB-JT Improved the Serum Biochemical Parameters in HFD-Fed Mice

**Figure 2:**
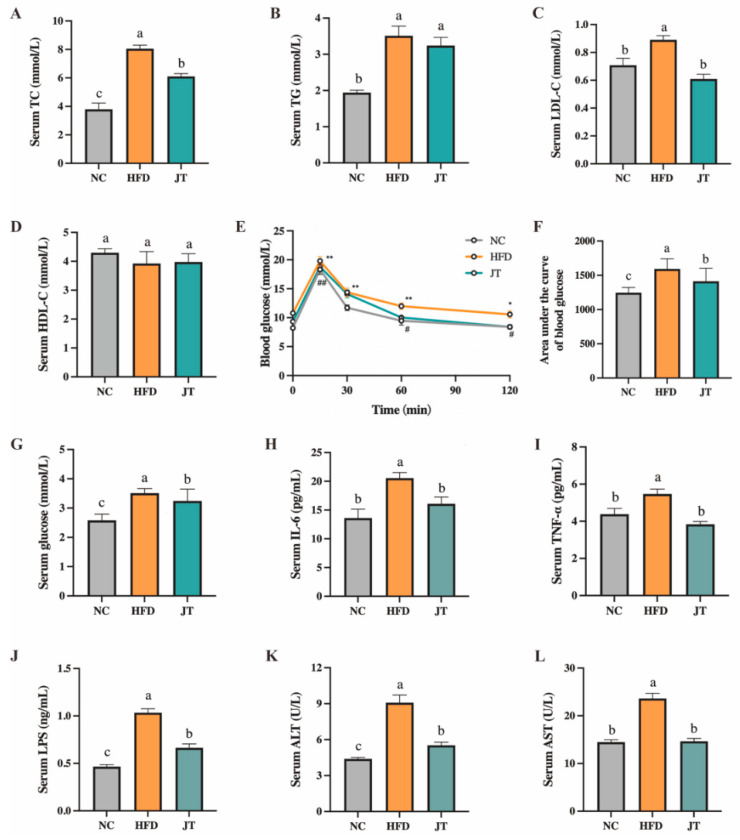
CB-JT improved the serum biochemical parameters in HFD-fed mice. (**A**) Serum TC. (**B**) Serum TG. (**C**) Serum LDL-C. (**D**) Serum HDL-C. (**E**) Blood glucose. (**F**) Area under the curve of blood glucose. (**G**) Serum glucose. (**H**) Serum LPS. (**I**) Serum IL-6. (**J**) Serum TNF-α. (**K**) Serum ALT. (**L**) Serum AST. NC: normal-chow group, HFD: high-fat diet group, JT: jasmine tea group. Data are expressed as the mean ± SEM. The mean value with different letters indicates significant differences (*p* < 0.05). HFD vs. NC: * *p* < 0.05, ** *p* < 0.01; JT vs. HFD: # *p* < 0.05, ## *p* < 0.01, ns for no significance.

Obesity is usually accompanied by altered plasma lipid profiles, inflammation, dysglycemia, endotoxemia as well as functional impairment of non-adipose tissue. As shown in Figure 2A–D, serum TC, TG and LDL-C levels were noticeably raised in the HFD group by 2.12, 1.81, and 1.26 times, respectively, compared with the NC group; CB-JT intervention reduced the levels of circulating TC, TG and LDL-C (*p* < 0.05, *p* > 0.05, *p* < 0.05). No evidence was found for significant variation in HDL levels among the NC, HFD, and JT groups. Figure 2E,F indicate that 60 min after the glucose injection, blood glucose values were significantly lowered by CB-JT, and the area under the curve (AUC) of blood glucose decreased in mice fed with CB-JT compared with the HFD group (*p* < 0.05). CB-JT treatment inhibited HFD-induced increment in fasting blood glucose levels (Figure 2G). These results indicated that HFD remarkably disrupted glucose homeostasis, while CB-JT markedly alleviated HFD-driven abnormal glucose metabolism. Furthermore, HFD significantly raised the circulating IL-6, TNF-α, and LPS levels (*p* < 0.05 for all analyses) compared with those of the NC group, while decreases in the endotoxin and inflammatory cytokine levels were observed in the JT group with statistical significance (Figure 2H,J). CB-JT was also proved to remarkably improve the increased levels of hepatic injury indicators, including AST and ALT in mice with HFD feeding (Figure 2K,L).

### 3.3. CB-JT Attenuated HFD-Induced Histological Injury

**Figure 3:**
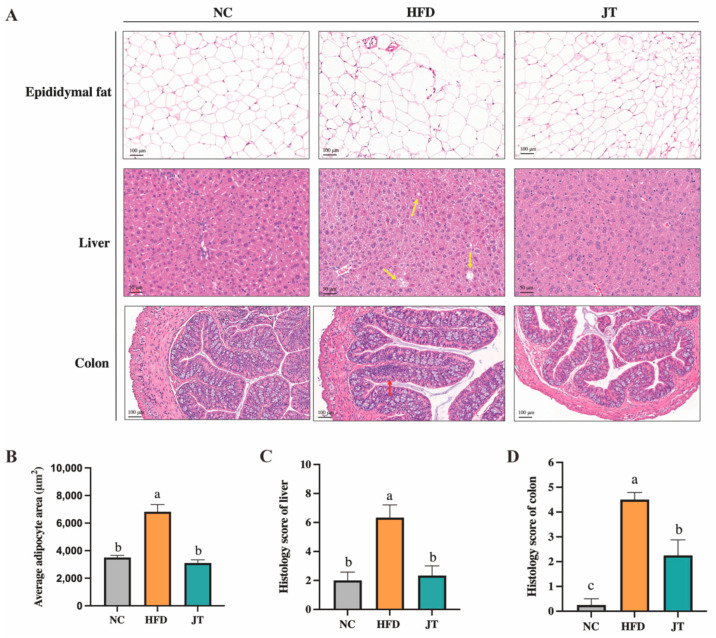
CB-JT attenuated HFD-induced histological injury. (**A**) Representative images of histological sections of epididymal fat, liver, and colon tissues. Yellow arrows: balloon-like structures in liver tissues; red arrows: inflammatory infiltration in colonic tissues. (**B**) Average adipocyte area of epididymis adipose. (**C**) Histology score of liver. (**D**) Histology score of colon tissues. NC: normal-chow group, HFD: high-fat diet group, JT: jasmine tea group. Data are expressed as the mean ± SEM. The mean value with different letters indicates significant differences (*p* < 0.05).

A histological examination was further performed to probe the preventive effect of CB-JT supplementation on HFD-induced fat accumulation and inflammation. As illustrated in Figure 3A,B, HFD feeding promoted adipocyte hypertrophy and raised adipocyte size compared to that in NC-fed mice (*p* < 0.05), while fat accumulation in HFD-mice was reversed by CB-JT intervention (*p* < 0.05). Additionally, liver histologic analysis suggested that significant hepatic lipid accumulation and balloon-like structures (yellow arrows) emerged in the HFD group, but these histological changes were effectively improved by CB-JT treatment. The histology score of liver tissue also showed that the hepatic injury was notably alleviated in the JT group (Figure 3C). Moreover, HFD feeding also induced inflammatory infiltration (red arrows) with intact crypt structures and similar numbers of crypt goblet cells, indicating low-grade inflammation in the HFD-fed group. CB-JT significantly reduced the inflammatory area and attenuated inflammatory cell infiltration. Histopathological scores of colon tissues demonstrated that CB-JT supplementation partially alleviated or completely reversed HFD-induced colonic histological damage (Figure 3D).

### 3.4. CB-JT Regulated HFD-Induced Abnormal Expression of Lipid Metabolism-Related Genes

**Figure 4:**
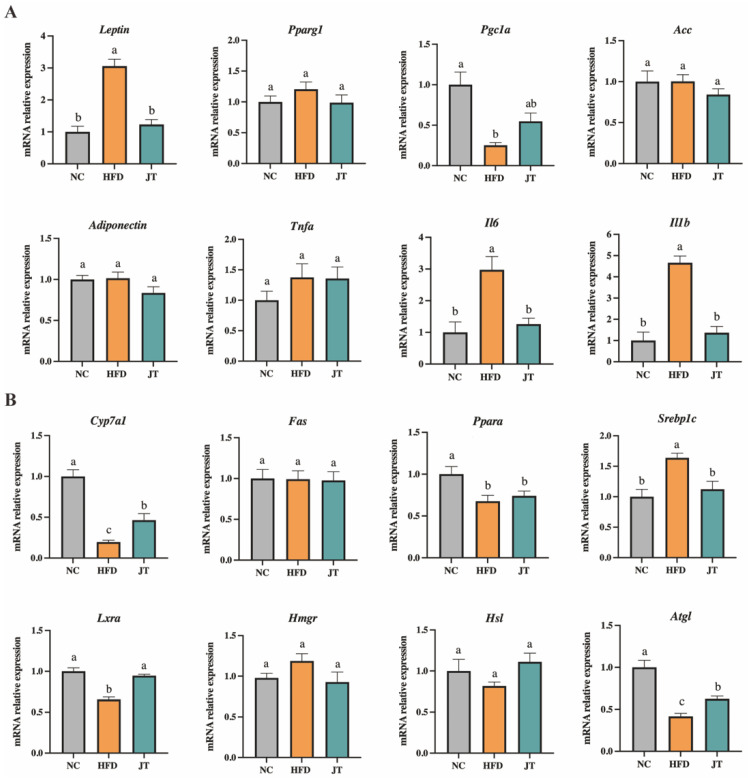
CB-JT regulated HFD-induced abnormal expression of lipid metabolism-related genes. (**A**) Relative mRNA expression in the epididymal adipose tissue. (**B**) Relative mRNA expression in the liver tissue. Expression levels were normalized to *β-ACTIN* and expressed as relative fold changes in comparison with the NC group (mean ± SEM). NC: normal-chow group, HFD: high-fat diet group, JT: jasmine tea group. The mean value with different letters indicates significant differences (*p* < 0.05).

To determine whether CB-JT treatment normalized lipogenesis and lipolysis at the gene transcript levels, some key genes for lipogenic mediators in liver and epididymal adipose tissues were next quantified. As shown in Figure 4A, HFD upregulated the mRNA levels of critical lipogenic genes (*Leptin*, *Il6*, and *Il1b*) by 3.06, 2.98 and 4.68 times, respectively, with significant difference in date compared to the NC group. A 0.25-fold decrease in the mRNA expression of *Pgc1a* was also noticed in adipose tissues compared to that in the NC group (*p* < 0.05). Figure 4B shows that the hepatic gene expression of *Cyp7a1*, *Lxra*, *Ppara*, and *Atgl* was significantly suppressed in mice fed with HFD, and the mRNA level of *Srebp1c* was promoted by HFD feeding compared to its respective level in NC-fed mice with statistical significance. However, CB-JT treatment significantly inhibited HFD-induced differential expression of *Leptin*, *Il6*, and *Il1b* in adipose tissue and *Cyp7a1*, *Lxra*, and *Atgl* in liver as well (*p* < 0.05 for all analyses).

### 3.5. CB-JT Modulated HFD-Induced Gut Microbiota Disorder

**Figure 5:**
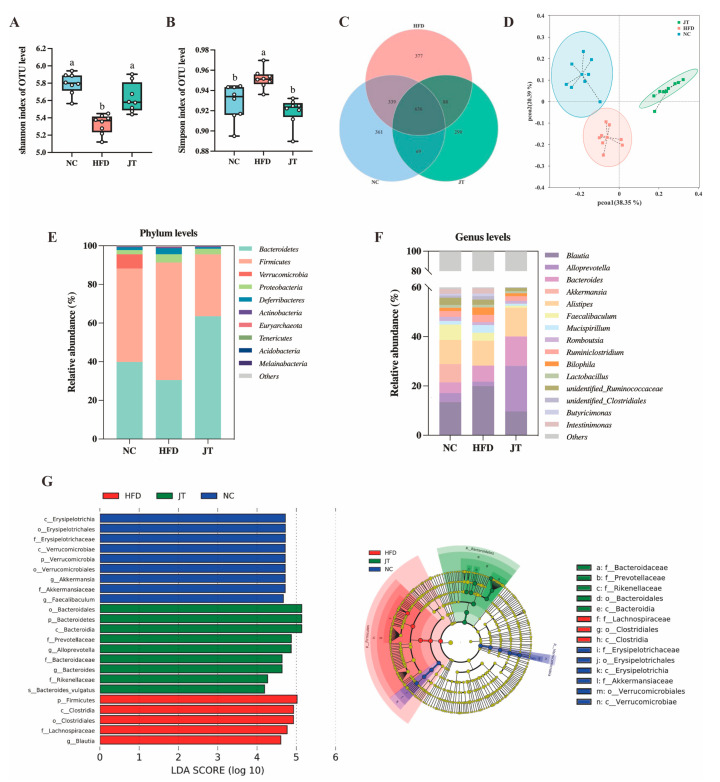
CB-JT modulated HFD-induced gut microbial disorder. (**A**) Shannon index. (**B**) Simpson index. (**C**) Venn diagrams of OTUs in the gut microbiota among groups. (**D**) PCoA. (**E**) Bacterial taxonomic composition at the phylum level. (**F**) Bacterial taxonomic composition at the genus level (**G**) LEfSe analysis (LDA score > 4). NC: normal-chow group, HFD: high-fat diet group, JT: jasmine tea group. Data are expressed as the mean ± SEM. The mean value with different letters indicates significant differences (*p* < 0.05).

Growing evidence has supported the correlation between gut microflora and metabolic diseases. Figure 5A,B shows that the Shannon and Simpson indices of the HFD group differed significantly from those of the mice on normal chow, whereas the changes in the corresponding parameters were remarkably inhibited by JT treatment and recovered to normal levels. The gut microflora composition in response to the different dietary interventions is shown in the Venn diagram (Figure 5C), where NC, HFD, and JT groups exhibited 361, 377, and 290 unique OTUs, respectively, and shared 636 overlapping OTUs. To understand the effect CB-JT on the gut microbiota structure, principal coordinate analysis (PCoA) was then conducted with PC1 of 38.35% and PC2 of 20.39% (Figure 5D). The graphs also signified that the gut bacterial structure in the NC, HFD, and JT groups was clustered separately and classified into three distinct clusters, implicating a dramatic impact of CB-JT on gut microbial composition in the HFD group.

The distinct differences in microbial composition between the three groups were next assessed at various phylum and genus levels. Figure 5E,F shows that the gut microflora was dominated by Bacteroidetes and Firmicutes, which is consistent with findings in the previous literature [19]. LEfSe analysis was then conducted to classify specific phylotypes that were significantly associated with HFD and CB-JT treatments. As illustrated in Figure 5G, a total of 23 OTUs were screened as phylogenetic types, whose relative abundance varied significantly with HFD feeding and CB-JT treatment. Pathogenic taxa, including *Lachnospiraceae*, *Firmicutes*, *Clostridiales*, and *Clostridia*, were enriched in the HFD group, in contrast with the mice on normal chow, while higher levels of some beneficial phylotypes including *Bacteroides*, and *Alloprevotella* were observed in the JT group.

**Figure 6:**
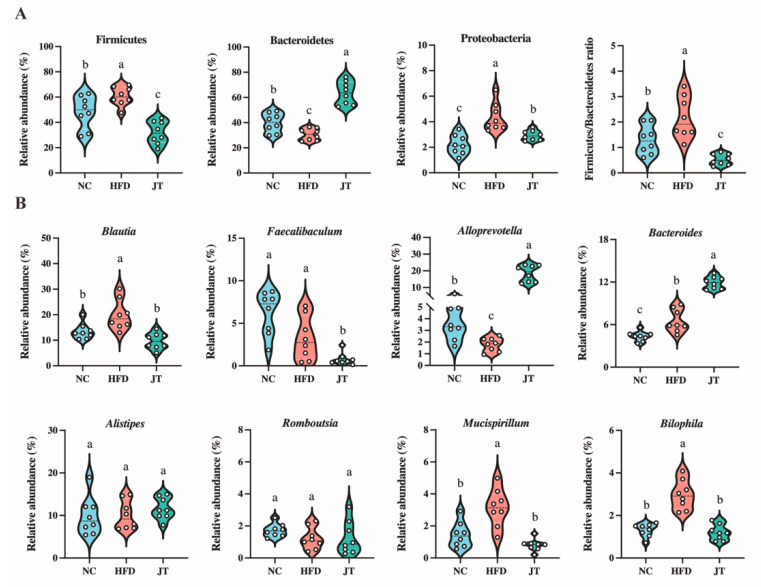
The abundance of predominant bacteria in intestine flora profiling of HFD-fed mice differed after CB-JT treatment. (**A**) Relative enrichment of top bacterial phyla and Firmicutes to Bacteroidetes (F/B) ratio. (**B**) Relative enrichment of dominant genera. NC: normal-chow group, HFD: high-fat diet group, JT: jasmine tea group. The mean value with different letters indicates significant differences (*p* < 0.05).

Specifically, the composition of phyla and the predominant genera (top 10 relative abundance) of the different treatments can be seen in Figure 6A,B. CB-JT treatment markedly lowered the abundance of Firmicutes and Proteobacteria as well as the ratio of Firmicutes to Bacteroidetes (F/B) in the HFD group with statistical differences. Additionally, HFD feeding inhibited the growth of *Alloprevotella* and enriched the abundance of *Blautia*, *Bacteroides*, *Mucispirillum*, and *Bilophila* in comparison to the levels found in the NC group (*p* < 0.05 for all analyses). In contrast, the significantly expressed genera including *Blautia*, *Alloprevotella*, *Mucispirillum*, and *Bilophila* were remarkably mitigated by CB-JT supplementation with statistical significance. The results demonstrated that CB-JT treatment reversed the HFD-driven abnormal abundance of specific bacteria and promoted the sustained enrichment of beneficial bacteria, thus regulating gut microbiota dysbiosis.

### 3.6. Correlation Analysis and Predictive Function Profiling of Gut Microbiota

**Figure 7:**
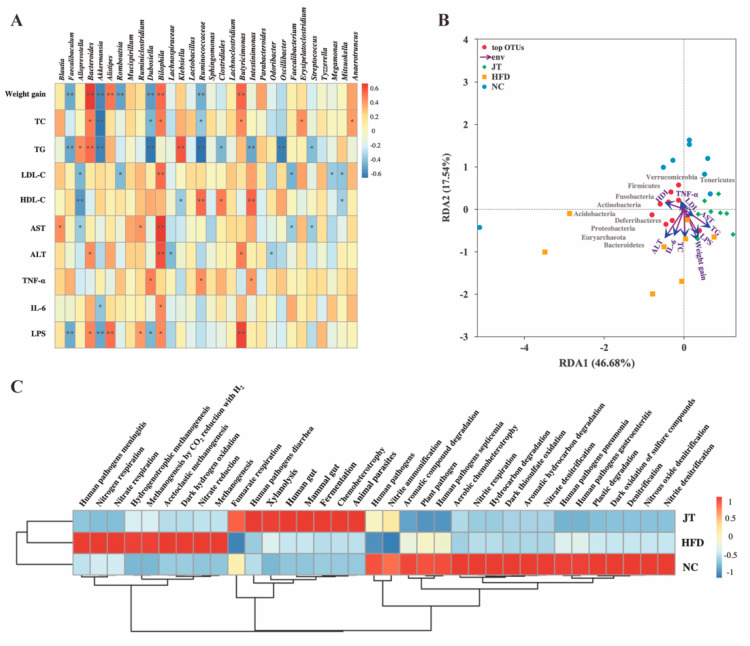
Correlation analysis of gut microbiota and obesity indicators. (**A**) Heatmap of Spearman’s correlation between gut microflora and obesity parameters in mice affected by HFD feeding or CB-JT treatment, * *p* < 0.05, ** *p* < 0.01. (**B**) RDA analysis. (**C**) Predictive function profiling of gut microbiota. NC: normal-chow group, HFD: high-fat diet group, JT: jasmine tea group.

The correlations between gut microbiota and obesity indicators were estimated with Spearman’s correlation analysis, and the results are illustrated in a heatmap (Figure 7A). Weight gain and the circulating concentrations of TC and TG were positively correlated with *Bacteriodes*, *Bilophila*, and *Butyricimonas*, and exhibited a negative correlation with *Faecalibaculum*, *Akkermansia*, and *Ruminococcaceae*. *Bilophila* was observed to be positively associated with the levels of AST, ALT, and IL-6, demonstrating that *Bilophila might* relate to HFD-induced hepatic injury and inflammatory development. RDA was applied to assess the correlation between environmental attributes and gut microbiota structure during CB-JT treatment in HFD-fed mice (Figure 7B). RDA1 explained 46.68% of the variation, while RDA2 represented 17.54% of the variation. The enriched area varied widely within the NC and HFD groups, whereas a slighter differential enrichment was observed with CB-JT treatment. According to previous study, vector arrows were labeled with the corresponding environmental factors, and the length of the arrows indicated the relative influence [20]. This finding showed that the CB-JT preventive effect on HFD-driven obesity might be related to serum lipid levels, endotoxin, and liver function. Moreover, the relative abundance of *Verrucomicrobia*, *Tenericutes*, *Firmicutes*, *Fusobacteria*, and *Actinobacteria* showed a negative association with obesity-related parameters such as blood lipids and weight gain, whereas *Acidobacteria*, *Deferribacters*, *Proteobacteria Euryarchaeota*, and *Bacteroidetes* exerted positive relationships with circulating IL-6 and ALT levels, contributing to hepatic injury and inflammation.

Functional annotation of prokaryotic taxa (FAPROTAX) analysis was applied to perform the functional prediction (Figure 7C). CB-JT intervention reverted the differentially enriched pathways related to pathogens, nitrogen respiration, nitrate respiration, hydrogenotrophic methanogenesis, methanogenesis, methanogenesis by CO_2_ reduction with H_2_, acetoclastic methanogenesis, dark hydrogen oxidation, and nitrate reduction caused by HFD feeding.

## 4. Discussion

Obesity is a worldwide metabolic disease linked to nonalcoholic fatty liver disease (NAFLD) and hypertension as well as various cancers. The excessive availability of sugar-sweetened drinks was associated with higher risk of overweight and obesity [21]. Tea beverages are known to contain a variety of polyphenols, polysaccharide, and theanine, which have strong anti-obesity properties [22]. Dietary supplementation with sugar-free functional tea alternatives is thus a feasible strategy for obesity prevention. A comparative study found that the dominant phenolics such as catechins and chlorogenic acid in jasmine/green tea exhibited higher levels than those in oolong tea and black tea [23]. Previously, jasmine tea epicatechins were found to exert hypolipidemic effects in HFD-fed hamsters, which is consistent with current findings in a mouse model [24]. Apart from dominant catechins, novel polysaccharide sub-fractions from jasmine tea were also proved to have anti-hyperglycemic and antioxidant capacity in a recent study, which further corroborates the health attributes of jasmine tea [13]. Meanwhile, considering the multiple advantages of cold-brewed jasmine tea, including better characteristic flavors, higher levels of unoxidized bioactive components, and portable convenience for future development, we thus speculate that CB-JT may support metabolic health as a widely consumed functional tea beverage [10,11]. In our study, a C57/BL6J obese model was established to examine the potential of CB-JT in preventing obesity and the molecular mechanism involved. The mice in the JT group were given ad libitum CB-JT to simulate the most commonly used pattern of tea consumption for the human body, and the daily consumption of dominant catechins in our animal experiment was approximately equivalent to the intake of 2 cups of tea a day in humans, according to the dose conversion criteria of body surface area [25]. As expected, food intake and water consumption were not notably affected by jasmine tea in a free-feeding context, while CB-JT at this free intake dose significantly alleviated HFD-driven weight gain, lipid deposition, and dyslipidemia, which was in line with former studies reported for green tea, black tea, and oolong tea [26]. The increased concentrations of TC might be associated with the excess mobilization of fatty acids from peripheral deposits to the liver, thus leading to hepatic steatosis [27]. The elevated AST and ALT levels also suggested hepatic injury caused by HFD feeding, and CB-JT significantly suppressed the increase in these indicators for NAFLD. HFD has been proven to cause impaired intestinal integrity and increase the leakage of microbiota-derived LPS, thus resulting in systemic inflammation that intensifies obesity pathogenesis [28]. Simultaneously, our findings showed that CB-JT attenuated metabolic endotoxemia, glucose metabolic dysfunction, and systemic inflammatory response, marked by lower levels of LPS, glucose tolerance and proinflammatory factors, respectively.

Liver and adipose tissue are essential organs for energy metabolism. A considerable amount of evidence has shown that obesity is usually accompanied with the abnormal expression of some critical genes involved in the processes of adipogenesis, lipogenesis, and lipolysis in liver and epididymal adipose tissues [29]. Some anti-obesity therapies have been reported to exert their efficacy against obesity by modulating gene expression involved in fatty acid biosynthesis and lipid metabolism [30]. CB-JT-mediated regulation (activation/repression) of differentially expressed genes driven by HFD feeding was then detected. *Cyp7a1* and *Atgl* usually are involved in bile acid synthesis, cholesterol accumulation and energy metabolism regulation [31,32]. *Ppara* can repress inflammation and reduce the expression of *Tnfa*, *Il6* and *Il1b* by inhibiting the nuclear factor κB pathway [33]. *Pgc1a* plays a key role in regulating insulin sensitivity as an essential transcriptional regulator in mitochondrial and metabolic processes [34]. *Leptin* is primarily responsible for regulating lipid metabolism, angiogenesis, insulin sensitivity and inflammatory processes [35]. *Lxra* shows well-defined roles in mediating lipogenesis and regulating glucose homeostasis [36]. *Srebp1c* contributes to lipid homeostasis and fatty acid synthesis [37]. In our study, HFD promoted significantly differential expression of *Cyp7a1*, *Lxra*, *Ppara*, *Atgl*, *Pgc1a*, *Leptin*, *Srebp1c*, *Il6*, and *Il1b*, whereas CB-JT inhibited the differential expression of *Lxra*, *Cyp7a1*, *Atgl*, *Srebp1c*, *Leptin*, *Il6*, and *Il1b.* This result suggests that CB-JT prevented the development of obesity profiling partially by regulating cholesterol accumulation, energy metabolism, insulin sensitivity, and inflammatory processes.

Functional components in tea beverages, such as polyphenols and theanine, are usually poorly absorbed, and thus the health benefits of tea beverages may be attributed to the interaction between these bioactive ingredients with gut microflora. Additionally, gut microbiota has been confirmed to regulate obesity traits recently [38]. We further hypothesized that CB-JT might prevent obesity partially by modulating gut flora. Our results show that gut microbial diversity in the JT-treated mice remained at a high level in comparison to that of the HFD group, which was consistent with the capacity of green tea to increase levels of gut microbiota diversity, as reported previously [39]. CB-JT also reverted HFD-induced higher levels of the F/B ratio, indicating an anti-obesity capacity as reported in [40]. Furthermore, the increased relative abundance of Proteobacteria has been reported to be associated with LPS release, recognized as an indicator of NAFLD mediated by the gut–liver axis [41]. Enriched growth of Proteobacteria was observed in the HFD group, while CB-JT noticeably inhibited the differential expression of Proteobacteria. *Blautia*, the most enriched genus, depicted a positive association with the levels of AST, serum lipids and inflammatory factors [42]. The relative abundance of *Faecalibaculum* and *Bacteroides* presented a contrary tendency during the HFD feeding and CB-JT treatment. *Faecalibaculum*, a pro-inflammatory bacterium, was raised by HFD feeding and positively correlated with inflammatory factors [43]. Conversely, *Bacteroides* was promoted by CB-JT treatment, presenting a potential for suppressing the release of cytokines and development of autoimmune diseases [44]. As reported, *Alloprevotella*, enriched in mice supplemented with CB-JT, is widely recognized as a beneficial bacterium for producing short-chain fatty acids (SCFAs) and protecting liver function [45]. *Bilophila* has also been documented to be involved in SCFA synthesis, adipocyte differentiation, lipoprotein hydrolysis, and lipid metabolism [46]. *Mucispirillum* has been considered to participate in energy metabolism and contribute to the generation of free fatty acids coupled with SCFAs [47]. Our results also indicated that CB-JT suppressed the relative abundance of *Bilophila* and *Mucispirillum*, which is consistent with the bacteriostatic effects of quercetin and resveratrol [48]. In the Spearman analysis, three strains of detrimental bacteria in HFD-mediated obesity were identified. *Butyricimonas* was positively correlated with weight gain and LPS release, which has been proven to involve the butyrate production pathway and inflammation [49]. *Erysipelotrichaceae* was also positively associated with the obesity index. A previous report has also shown that *Erysipelotrichaceae* may be enriched with HFD feeding via the extraction of more energy from the diet, and thus re-shape the lipid profiles and exacerbate obesity [50].

In our study, cold-brewed jasmine tea was found to neutralize the HFD-induced adverse effects on lipid metabolism and gut microbiota profile. Our findings might provide a novel perspective for the prevention of obesity and related metabolic diseases. Nevertheless, further clinical validation is required to evaluate the long-term efficacy of cold-brewed jasmine tea and develop a supportive nutritional recommendation.

## 5. Conclusions

In conclusion, this study indicated that cold-brewed jasmine tea attenuated weight gain, abnormal serum blood levels, fat accumulation, inflammation, glucose intolerance, metabolic endotoxemia, as well as differentially expressed genes related to lipid metabolism. CB-JT treatment also improved the gut microbial diversity, reshaped the gut microbial composition, promoted beneficial bacteria such as *Alloprevotella* and *Bacteroides*, and inhibited the growth of pathogenic bacterial taxa including *Blautia*, *Bilophila* and *Mucispirillum.* Our findings offer evidence that cold brewed-jasmine tea could serve as a functional tea beverage resource for the prevention of obesity and gut microbiome dysbiosis.

## Data Availability

Not applicable.

## Supplementary Materials

The following supporting information can be downloaded at: https://www.mdpi.com/article/10.3390/nu14245359/s1, Table S1: The main constituents of cold-brewed jasmine tea; Table S2: Primer sequences for qPCR.

## References

[1] Wang J., Wang P., Li D., Hu X., Chen F. (2019). Beneficial effects of ginger on prevention of obesity through modulation of gut microbiota in mice. Eur. J. Nutr..

[2] Anhê F.F., Nachbar R.T., Varin T.V., Trottier J., Dudonné S., Le Barz M., Feutry P., Pilon G., Barbier O., Desjardins Y. (2019). Treatment with camu camu (Myrciaria Dubia) prevents obesity by altering the gut microbiota and increasing energy expenditure in diet-induced obese mice. Gut.

[3] Ye J., Zhao Y., Chen X., Zhou H., Yang Y., Zhang X., Huang Y., Zhang N., Lui E.M.K., Xiao M. (2021). Pu-erh tea ameliorates obesity and modulates gut microbiota in high fat diet fed mice. Food Res. Int..

[4] Aravind S.M., Wichienchot S., Tsao R., Ramakrishnan S., Chakkaravarthi S. (2021). Role of dietary polyphenols on gut microbiota, their metabolites and health benefits. Food Res. Int..

[5] Sainsbury E., Magnusson R., Thow A.-M., Colagiuri S. (2020). Explaining resistance to regulatory interventions to prevent obesity and improve nutrition: A case-study of a sugar-sweetened beverages tax in Australia. Food Policy.

[6] Muller M., De Beer D., Truzzi C., Annibaldi A., Carloni P., Girolametti F., Damiani E., Joubert E. (2020). Cold brewing of rooibos tea affects its sensory profile and physicochemical properties compared to regular hot, and boiled brewing. LWT.

[7] Verbeke W. (2006). Functional foods: Consumer willingness to compromise on taste for health?. Food Qual. Prefer..

[8] Lin S.-D., Yang J.-H., Hsieh Y.-J., Liu E.-H., Mau J.-L. (2014). Effect of different brewing methods on quality of green tea. J. Food Process. Preserv..

[9] Damiani E., Bacchetti T., Padella L., Tiano L., Carloni P. (2014). Antioxidant activity of different white teas: Comparison of hot and cold tea infusions. J. Food Compos. Anal..

[10] Jiang J. (2019). The hypoglycemic effect of green tea brewed in cold water is more effective. Mol. Nutr. Food Res..

[11] Ito Y., Sugimoto A., Kakuda T., Kubota K. (2002). Identification of potent odorants in Chinese jasmine green tea scented with flowers of jasminum sambac. J. Agric. Food Chem..

[12] An H., Ou X., Zhang Y., Li S., Xiong Y., Li Q., Huang J., Liu Z. (2022). Study on the key volatile compounds and aroma quality of jasmine tea with different scenting technology. Food Chem..

[13] Tang Y., Sheng J., He X., Sun J., Wei Z., Liu G., Li C., Lin B., Li L. (2021). Novel antioxidant and hypoglycemic water-soluble polysaccharides from jasmine tea. Foods.

[14] Kuroda K., Inoue N., Ito Y., Kubota K., Sugimoto A., Kakuda T., Fushiki T. (2005). Sedative effects of the jasmine tea odor and (R)-(−)-linalool, one of its major odor components, on autonomic nerve activity and mood states. Eur. J. Appl. Physiol..

[15] Zhu J., Cai R., Tan Y., Wu X., Wen Q., Liu Z., Ouyang S.-H., Yin Z., Yang H. (2020). Preventive consumption of green tea modifies the gut microbiota and provides persistent protection from high-fat diet-induced obesity. J. Funct. Foods.

[16] Yoo A., Kim M.J., Ahn J., Jung C.H., Seo H.D., Ly S.Y., Ha T.Y. (2022). Fuzhuan brick tea extract prevents diet-induced obesity via stimulation of fat browning in mice. Food Chem..

[17] Wang S., Zeng T., Zhao S., Zhu Y., Feng C., Zhan J., Li S., Ho C.-T., Gosslau A. (2022). Multifunctional health-promoting effects of oolong tea and its products. Food Sci. Hum. Wellness.

[18] Wang C., Lv S.D., Wang J.X., Qiu X.L., Wu Y.S., Meng Q.X. (2017). Processing technologies affect the aroma but not the taste of teas: A study of Yunnan Biluochun, Jiangsu Biluochun, and other regular green teas. Int. J. Food Prop..

[19] Wen J., Ma L., Xu Y., Wu J., Yu Y., Peng J., Tang D., Zou B., Li L. (2020). Effects of probiotic litchi juice on immunomodulatory function and gut microbiota in mice. Food Res. Int..

[20] Mondal R., Bhat A. (2020). Temporal and environmental drivers of fish-community structure in tropical streams from two contrasting regions in India. PLoS ONE.

[21] Anari R., Amani R., Veissi M. (2017). Sugar-sweetened beverages consumption is associated with abdominal obesity risk in diabetic patients. Diabetes Metab. Syndr. Clin. Res. Rev..

[22] Zhang X., Zhang M., Ho C.-T., Guo X., Wu Z., Weng P., Yan M., Cao J. (2018). Metagenomics analysis of gut microbiota modulatory effect of green tea polyphenols by high fat diet-induced obesity mice model. J. Funct. Foods.

[23] Vu D.C., Alvarez S. (2021). Phenolic, carotenoid and saccharide compositions of vietnamese camellia sinensis teas and herbal teas. Molecules.

[24] Chan P.T., Fong W.P., Cheung Y.L., Huang Y., Ho W.K.K., Chen Z.Y. (1999). Jasmine green tea epicatechins are hypolipidemic in hamsters (*Mesocricetus auratus*) fed a high fat diet. J. Nutr..

[25] Reagan-Shaw S., Nihal M., Ahmad N. (2008). Dose translation from animal to human studies revisited. FASEB J..

[26] Sun L., Xu H., Ye J., Gaikwad N.W. (2019). Comparative effect of black, green, oolong, and white tea intake on weight gain and bile acid metabolism. Nutrition.

[27] Katsiki N., Mikhailidis D.P., Mantzoros C.S. (2016). Non-alcoholic fatty liver disease and dyslipidemia: An update. Metabolism.

[28] Chen P.B., Black A.S., Sobel A.L., Zhao Y., Mukherjee P., Molparia B., Moore N.E., Aleman Muench G.R., Wu J., Chen W. (2020). Directed remodeling of the mouse gut microbiome inhibits the development of atherosclerosis. Nat. Biotechnol..

[29] Huang J., Zhou Y., Wan B., Wang Q., Wan X. (2017). Green tea polyphenols alter lipid metabolism in the livers of broiler chickens through increased phosphorylation of AMP-activated protein kinase. PLoS ONE.

[30] Suk S., Kwon G.T., Lee E., Jang W.J., Yang H., Kim J.H., Thimmegowda N.R., Chung M.-Y., Kwon J.Y., Yang S. (2017). Gingerenone A, a polyphenol present in ginger, suppresses obesity and adipose tissue inflammation in high-fat diet-fed mice. Mol. Nutr. Food Res..

[31] Duan Y., Zhang F., Yuan W., Wei Y., Wei M., Zhou Y., Yang Y., Chang Y., Wu X. (2019). Hepatic cholesterol accumulation ascribed to the activation of ileum Fxr-Fgf15 pathway inhibiting hepatic Cyp7a1 in high-fat diet-induced obesity rats. Life Sci..

[32] Attané C., Peyot M.-L., Lussier R., Poursharifi P., Zhao S., Zhang D., Morin J., Pineda M., Wang S., Dumortier O. (2016). A beta cell ATGL-lipolysis/adipose tissue axis controls energy homeostasis and body weight via insulin secretion in mice. Diabetologia.

[33] Massaro M., Scoditti E., Pellegrino M., Carluccio M.A., Calabriso N., Wabitsch M., Storelli C., Wright M., De Caterina R. (2016). Therapeutic potential of the dual peroxisome proliferator activated receptor (PPAR)α/γ agonist aleglitazar in attenuating TNF-α-mediated inflammation and insulin resistance in human adipocytes. Pharmacol. Res..

[34] Santos J.M., Tewari S., Benite-Ribeiro S.A. (2014). The effect of exercise on epigenetic modifications of PGC1: The impact on type 2 Diabetes. Med. Hypotheses.

[35] Montserrat-de la Paz S., Pérez-Pérez A., Vilariño-García T., Jiménez-Cortegana C., Muriana F.J.G., Millán-Linares M.C., Sánchez-Margalet V. (2021). Nutritional modulation of leptin expression and leptin action in obesity and obesity-associated complications. J. Nutr. Biochem..

[36] Matsusue K., Aibara D., Hayafuchi R., Matsuo K., Takiguchi S., Gonzalez F.J., Yamano S. (2014). Hepatic PPARγ and LXRα independently regulate lipid accumulation in the livers of genetically obese mice. FEBS Lett..

[37] Lee G., Kim Y.Y., Jang H., Han J.S., Nahmgoong H., Park Y.J., Han S.M., Cho C., Lim S., Noh J.-R. (2022). SREBP1c-PARP1 axis tunes anti-senescence activity of adipocytes and ameliorates metabolic imbalance in obesity. Cell Metab..

[38] Li S.-Z., Zeng S.-L., Liu E.H. (2022). Anti-obesity natural products and gut microbiota. Food Res. Int..

[39] Pérez-Burillo S., Navajas-Porras B., López-Maldonado A., Hinojosa-Nogueira D., Pastoriza S., Rufián-Henares J.Á. (2021). Green tea and its relation to human gut microbiome. Molecules.

[40] Magne F., Gotteland M., Gauthier L., Zazueta A., Pesoa S., Navarrete P., Balamurugan R. (2020). The firmicutes/bacteroidetes ratio: A relevant marker of gut dysbiosis in obese patients?. Nutrients.

[41] Vasques-Monteiro I.M.L., Silva-Veiga F.M., Miranda C.S., de Andrade Gonçalves É.C.B., Daleprane J.B., Souza-Mello V. (2021). A rise in proteobacteria is an indicator of gut-liver axis-mediated nonalcoholic fatty liver disease in high-fructose-fed adult mice. Nutr. Res..

[42] Wu C., Zhao Y., Zhang Y., Yang Y., Su W., Yang Y., Sun L., Zhang F., Yu J., Wang Y. (2022). Gut microbiota specifically mediates the anti-hypercholesterolemic effect of berberine (BBR) and facilitates to predict BBR’s cholesterol-decreasing efficacy in patients. J. Adv. Res..

[43] Cai W., Xu J., Li G., Liu T., Guo X., Wang H., Luo L. (2020). Ethanol extract of propolis prevents high-fat diet-induced insulin resistance and obesity in association with modulation of gut microbiota in mice. Food Res. Int..

[44] Zhao R., Hu Q., Ma G., Su A., Xie M., Li X., Chen G., Zhao L. (2019). Effects of flammulina velutipes polysaccharide on immune response and intestinal microbiota in mice. J. Funct. Foods.

[45] Hu R., Guo W., Huang Z., Li L., Liu B., Lv X. (2018). Extracts of ganoderma lucidum attenuate lipid metabolism and modulate gut microbiota in high-fat diet fed rats. J. Funct. Foods.

[46] Olson C.A., Iñiguez A.J., Yang G.E., Fang P., Pronovost G.N., Jameson K.G., Rendon T.K., Paramo J., Barlow J.T., Ismagilov R.F. (2021). Alterations in the gut microbiota contribute to cognitive impairment induced by the ketogenic diet and hypoxia. Cell Host Microbe.

[47] Chen S., Xu M., Zhou M., He Y., Li Y., Lang H., Wei X., Yan L., Xu H. (2022). *Hibiscus manihot* L. improves obesity in mice induced by a high-fat diet. J. Funct. Foods.

[48] Zhao L., Zhang Q., Ma W.N., Tian F., Shen H.Y., Zhou M.M. (2017). A combination of quercetin and resveratrol reduces obesity in high-fat diet-fed rats by modulation of gut microbiota. Food Funct..

[49] Cantoni C., Lin Q., Dorsett Y., Ghezzi L., Liu Z., Pan Y., Chen K., Han Y., Li Z., Xiao H. (2022). Alterations of host-gut microbiome interactions in multiple sclerosis. eBioMedicine.

[50] Bailén M., Bressa C., Martínez-López S., González-Soltero R., Montalvo Lominchar M.G., San Juan C., Larrosa M. (2020). Microbiota features associated with a high-fat/low-fiber diet in healthy adults. Front. Nutr..

