# Supplementary material for: Cold-Brewed Jasmine Tea Attenuates High-Fat Diet-Induced Obesity and Gut Microbial Dysbiosis
Source: Nutrients. 2022 Dec 16;14(24):5359. doi: 10.3390/nu14245359 (PMC9784320; doi:10.3390/nu14245359)
Supplement: Supplementary file 1 [file nutrients-14-05359-s001.zip › nutrients-2044031-supplementary.pdf]

1. HPLC analysis

The measurement was performed in HPLC system with an Agilent ZORBAX SB-AQ column (250 × 4.6 mm, 5μm) at 35 °C. The mobile phase was composed of water with 0.2% (v/v) acetic acid (A) and acetonitrile (B) with a linear gradient elution, 0-15min: 3%-10% B; 15-20min: 10%-20% B; 15-20min: 10%-20% B; 20-25min: 20%-25% B; 25-30min: 25%-30% B; 30-35min: 30%-35% B; 35-40min: 35%-3% B; 40-45min: 3%B. The samples were eluted at 1 mL/min flow rate and monitored at 270 nm. Each sample was performed for three independent extractions and the results can be found in Table S1.

Table S1. The main compositions of cold-brewed jasmine tea

Ingredients	Content
Polysaccharide	498.16±6.80 μg /mL
Theanine	49.4±0.64 μg /mL
Total phenolic content	415.11±8.67 μg GAE/mL
Catechins	μg /mL
EC	400.94±2.75
GC	63.2±2.02
GCG	48.83±1.43
EGCG	36.07±0.73
EGC	24.14±0.62
C	18.26±0.63
CG	13.31±0.24
ECG	12.94±0.37
Gallic acid	9.87±0.25

EC: epicatechin, GC: gallocatechin, GCG: gallocatechin gallate, EGCG: epigallocatechin gallate, EGC: epigallocatechin, C: catechin, CG: catechin gallate, ECG: epicatechin gallate.

2. Quantification of host gene expression

Table S2 Primer sequences for qPCR

Gene	Forward primer（5’→3’）	Reverse primer（5’→3’）
Leptin	CCTGTGGCTTTGGTCCTATCTG	AGGCAAGCTGGTGAGGATCTG
Pparg1	CCAGCATTTCTGCTCCACAC	ATTCTTGGAGCTTCAGGCCA
Pgc1a	AGCCGTGACCACTGACAACGAG	GCTGCATGGTTCTGAGTGCTAAG
Acc	GGCAGCAGTTACACCACATAC	TCATTACCTCAATCTCAGCATAGC
Adiponectin	CCCTGGTCTCCACGACTCTT	GCGAATATTGTGAAGCCCCC
Tnfa	AATGGCCTCCCTCTCATCAG	CCACTTGGTGGTTTGCTACG
Il6	ACTTCCATCCAGTTGCCTTCTTG	TGTTGGGAGTGGTATCCTCTGTG
Il1b	AAGGGCTG TTCCAAACCTTTGAC	TGCCTGAAGCT TTGTTGATGTGC
Cyp7a1	AACAACCTGCCAGTACTAGATAGC	GTGTAGAGTGAAGTCCTCCTTAGC
Fas	GCTGCGGAAACTTCAGGAAAT	AGAGACGTGTCACTCCTGGACTT
Ppara	TGCAGCCTCAGCCAAGTTGAA	TCCCGAACTTGACCAGCCA
Srebp1c	CTGGTGAGTGGAGGGACCAT	GACCGGTAGCGCTTCTCAAT
Lxra	TCAGAAGAACAGATCCGCTTG	CGCCTGTTACACTGTTGCT
Hmgr	TGCCTGGATGGGAAGGAGTA	GCACCTCCACCAAGGCTTAT
Hsl	GCTAGCCAGGCTCATCTCCT	GTTCTTGAGGTAGGGCTCGT
Atgl	ACAGCTCCAACATCCAC	AGCCCTGTTTGCACATCTCT
β-actin	ACAGCAGTTGGTTGGAGCAA	ACGCGACCATCCTCCTCTTA

Pparg1, peroxisome proliferator-activated receptor γ1; *Pgc1a*, peroxisome proliferator-activated receptor c coactivator 1α; *Acc*, acetyl-CoA carboxylase; Tnfa, tumor necrosis factor α; *Il6*, interleukin- 6; *Il1b*, interleukin- 1β; *Cyp7a1*, cholesterol 7α-hydroxylase; *Fas*, fatty acid synthase; *Ppara*, peroxisome proliferator-activated receptor alpha; *Srebp1c,* sterol regulatory element-binding protein-1c; *Lxra*, liver X receptors α; *Hmgr*, 3-hydroxy-3-methyl glutaryl coenzyme A reductase; *Hsl*, hormone-sensitive lipase; *Atgl*, adipose triglyceride lipase;
